# Updated Australian consensus statement on management of inherited bleeding disorders in pregnancy

**DOI:** 10.5694/mja2.50123

**Published:** 2019-03-29

**Authors:** Scott Dunkley, Julie A Curtin, Anthony J Marren, Robert P Heavener, Simon McRae, Jennifer L Curnow

**Affiliations:** ^1^ Institute of Haematology Royal Prince Alfred Hospital Sydney NSW; ^2^ The Children's Hospital at Westmead Sydney NSW; ^3^ Australian Haemophilia Centres Directors’ Organisation Melbourne VIC; ^4^ Royal Prince Alfred Hospital Sydney NSW; ^5^ Royal Adelaide Hospital Adelaide SA; ^6^ Haemophilia Treatment Centre Westmead Hospital Sydney NSW

**Keywords:** Pregnancy, Blood platelet disorders, Coagulation disorders, Transfusion medicine, Prenatal diagnosis, Postpartum, Genetic counseling

## Abstract

**Introduction:**

There have been significant advances in the understanding of the management of inherited bleeding disorders in pregnancy since the last Australian Haemophilia Centre Directors’ Organisation (AHCDO) consensus statement was published in 2009. This updated consensus statement provides practical information for clinicians managing pregnant women who have, or carry a gene for, inherited bleeding disorders, and their potentially affected infants. It represents the consensus opinion of all AHCDO members; where evidence was lacking, recommendations have been based on clinical experience and consensus opinion.

**Main recommendations:**

During pregnancy and delivery, women with inherited bleeding disorders may be exposed to haemostatic challenges. Women with inherited bleeding disorders, and their potentially affected infants, need specialised care during pregnancy, delivery, and postpartum, and should be managed by a multidisciplinary team that includes at a minimum an obstetrician, anaesthetist, paediatrician or neonatologist, and haematologist. Recommendations on management of pregnancy, labour, delivery, obstetric anaesthesia and postpartum care, including reducing and treating postpartum haemorrhage, are included. The management of infants known to have or be at risk of an inherited bleeding disorder is also covered.

**Changes in management as a result of this statement:**

Key changes in this update include the addition of a summary of the expected physiological changes in coagulation factors and phenotypic severity of bleeding disorders in pregnancy; a flow chart for the recommended clinical management during pregnancy and delivery; guidance for the use of regional anaesthetic; and prophylactic treatment recommendations including concomitant tranexamic acid.

Inherited bleeding disorders pose a unique challenge during pregnancy and childbirth. Women who are affected with, or are carriers of, inherited bleeding disorders, and their infants, are at risk of bleeding complications from haemostatic challenges during pregnancy and childbirth. Therefore, pregnancy and delivery need to be managed appropriately.^1^, ^2^, ^3^, ^4^, ^5^, ^6^

This statement is an update of the 2009 Australian Haemophilia Centre Directors’ Organisation (AHCDO) consensus statement on pregnancy and delivery management in women with inherited bleeding disorders.^7^ Over the past 9 years there have been key advances in the understanding and management of inherited bleeding disorders in pregnancy. The aim of this updated consensus statement is to improve the care of women who have, or are carriers of, inherited bleeding disorders, and their potentially affected infants, by providing practical recommendations for the management of pregnancy and delivery.

Despite inclusion of pregnancy and delivery management in a number of published evidence‐based guidelines on the management of inherited bleeding disorders, many guidelines are lengthy and not user‐friendly.^1^, ^2^, ^6^, ^8^, ^9^, ^10^

AHCDO includes all directors of haemophilia centres (adult and paediatric) throughout Australia, as well as additional haematologists registered through AHCDO who care for patients with haemophilia either at these sites or at satellite centres. The consensus statement was developed by an AHCDO working party following extensive consultation, face‐to‐face meetings and revisions. The final document represents the consensus opinion of all AHCDO members.

As pregnancy and delivery management for women with inherited bleeding disorders involves a multidisciplinary team that includes an obstetrician, anaesthetist, paediatrician or neonatologist, and haematologist, we anticipate that this consensus statement will serve as a useful reference document for the broader medical community.

## Methods

We reviewed current international clinical practice guidelines on the management of inherited bleeding disorders in pregnancy. We also conducted broad literature searches and manually searched reference lists. Inclusion criteria were restricted to high quality meta‐analyses, systematic reviews, randomised controlled trials and clinical practice guidelines. Studies published in English and conducted in humans, and with an abstract available, were included for review. In several areas, evidence was lacking and, in such cases, recommendations were based on the clinical experience and consensus opinion of the AHCDO directors.

## Inherited bleeding disorders in women

The most common inherited bleeding disorders and their phenotypes in women are outlined in Box 1.^1^, ^2^, ^4^, ^8^, ^11^

**Box 1:** Bleeding disorders, coagulation factor affected and expected range of bleeding phenotypes in women^1^, ^2^, ^4^, ^8^, ^11^, ^15^

Disorder	Coagulation factor affected	Bleeding phenotype in women*	Expected factor level changes during pregnancy	Treatment options (if required)
Haemophilia A	Factor VIII levels decreased†	Mild to moderate when levels < 40 IU/dL‡	Typically, increase throughout pregnancy	Factor VIII or DDAVP antenatally; factor VIII replacement therapy before delivery, if required
Haemophilia B	Factor IX levels decreased	Mild to moderate when levels < 40 IU/dL‡	No	Factor IX replacement therapy before delivery, if required
Factor XI deficiency	Factor XI levels decreased	Highly variable; risk increased with levels < 15 IU/dL	No	Factor XI replacement or fresh frozen plasma before delivery, if required
von Willebrand disease				
Type 1	VWF levels decreased†	Mild to moderate	Yes, tends to increase throughout pregnancy	VWF containing concentrates or DDAVP antenatally; VWF‐containing concentrates before delivery, if required
Type 2§	Dysfunctional VWF	Variable, usually moderate	No, small increases only§	VWF‐containing concentrates antenatally and before delivery, if required
Type 3	VWF absent	Severe (VWF antigen undetectable; factor VIII levels < 10 IU/dL)	No, does not improve	VWF‐containing concentrates antenatally and before delivery, if required
Rare coagulation deficiencies	Afibrinogenaemia; factor II, factor V, combined factor V and VIII, factor VII, factor X and factor XIII deficiencies	Highly variable, mild to severe, not always predictable based on factor levels Recurrent fetal loss associated with factor I (fibrinogen), factor II and factor XIII deficiencies	No	Specific factor replacement or fresh frozen plasma for factor V deficiency, if required
Congenital platelet disorders				
Glanzmann thrombasthenia	Disorder of platelet function	Often associated with severe bleeding phenotype	No	Avoid blood or platelet transfusion during pregnancy; HLA‐matched platelets transfused at delivery
Bernard–Soulier disease	Glycoprotein Ib‐IX‐V receptor abnormality	Often associated with severe bleeding phenotype	No	Avoid blood or platelet transfusion during pregnancy; HLA‐matched platelets transfused at delivery
Other		Usually mild	No	

DDAVP = D‐amino D‐arginine vasopressin (desmopressin); HLA = human leukocyte antigen; VWF = von Willebrand factor. * Bleeding risk factor levels: severe, < 1 IU/dL; moderately severe, 3–10 IU/dL; mild, 10–40 IU/dL; low, > 40 IU/dL. Women with levels < 1 IU/dL (severe) should be monitored more closely. † Levels may normalise during pregnancy.^6^ ‡ Women with factor VIII or IX levels > 40 IU/dL but below the lower limit of the reference interval (50–200 IU/dL, but note that laboratory reference intervals vary based on methodology) may also have increased bleeding tendencies.^5^^§^ Thrombocytopenia associated with type 2B von Willebrand disease may worsen during pregnancy. Note regarding units: 1 international unit (IU) of factor VIII activity is equivalent to that quantity of factor VIII in 1 mL of normal human plasma. Levels are cited in % or IU/dL as the two units are equivalent and can be used interchangeably. Some laboratories give units as IU/mL; note, 50 IU/dL = 50% = 0.5 IU/mL. ◆

Haemophilia A and B are X chromosome‐linked conditions that are often mistakenly thought to affect males with females being carriers; however, a range of phenotypes are observed in females who have one copy of an abnormal gene for coagulation factor VIII (haemophilia A) or factor IX (haemophilia B). This phenotype range extends from a carrier with normal factor levels (the majority of patients), to those with mildly reduced factor levels (mild haemophilia) through to (very rarely) those with extremely low factor levels consistent with severe haemophilia. This range is due to genetic factors including skewed X chromosome inactivation (lyonisation), Turner syndrome or other chromosomal translocations and deletions.

Although there are differences in the reference ranges cited by coagulation laboratories, the normal plasma range for both factor VIII and factor IX is 50–200 IU/dL.^6^ In practical terms, normal equates to levels of factor VIII, factor IX and von Willebrand factor antigen > 50 IU/dL for haemophilia A, haemophilia B and type 1 von Willebrand disease. Bleeding risk is dependent on factor levels and the lower the factor level below 40 IU/dL the greater the bleeding risk.^5^ Together, haemophilia A and B, von Willebrand disease, and factor VII and factor XI deficiency account for around 90% of all women with inherited bleeding disorders.^12^

## Physiological response expected in pregnancy

As part of the normal physiological response, factor VIII and von Willebrand factor antigen levels rise during pregnancy, usually reaching a plateau at around 29–35 weeks’ gestation (Box 1).^13^, ^14^

For many but not all women with low factor VIII levels due to a mutation in one of their factor VIII genes, this physiological rise in factor VIII will result in levels rising to within the normal range and thus there is no increased risk of bleeding. For some women, however, factor VIII levels may remain low at term.^6^, ^8^, ^13^, ^14^, ^15^ Factor IX and factor XI do not change significantly in pregnancy.^6^, ^8^, ^10^, ^15^, ^16^, ^17^, ^18^

For women with von Willebrand disease, the type influences their ability to mount this physiological response. Women with type 1 von Willebrand disease — the common, mild form — will achieve normal levels of von Willebrand factor antigen and factor VIII. Those with type 2, in which the function of the von Willebrand factor molecule is impaired, will show a rise in their von Willebrand factor antigen level, but the functional activity will remain low. Patients with type 2B may show worsening thrombocytopenia due to increased levels of the abnormal von Willebrand factor molecule. Patients with type 3 will not exhibit a rise in levels. Therefore, patients with type 2 and 3 von Willebrand disease typically remain at risk of bleeding.^16^

The rise in factor levels is unpredictable during pregnancy and ideally levels should be checked before pregnancy, at the first antenatal visit (Box 2),^19^ before any invasive procedure, and during the third trimester to facilitate planning of delivery.^1^, ^2^, ^6^, ^8^, ^14^, ^17^ After delivery, factor levels usually return to baseline after 7–21 days but may drop earlier.^5^, ^6^, ^8^, ^13^, ^14^, ^15^, ^16^, ^20^

**Box 2:**
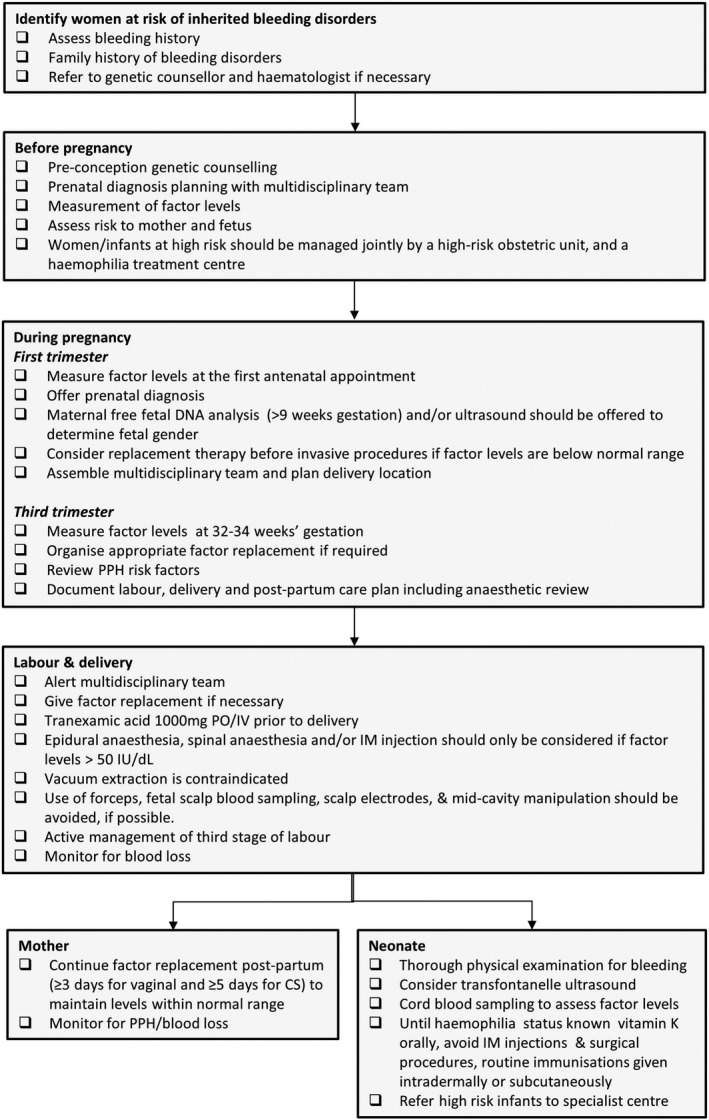
Key points in the care of women with inherited bleeding disorders and their potentially affected infants Adapted from Lavee and Kidson‐Gerber.^19^ CS = caesarean section; IM = intramuscular; IU = international units; IV = intravenous; PPH = postpartum haemorrhage; PO = per oral. ◆

## Management of pregnancy and delivery

### Before pregnancy

Pre‐conception genetic counselling should be considered for any mother at risk of having a child with haemophilia (eg, known family history, known low factor levels) to allow for appropriate planning with a multidisciplinary team (Box 2). If factor levels are not known, they should be measured and, if levels are low, the pregnancy should be managed appropriately. Historical diagnosis and response to desmopressin (D‐amino D‐arginine vasopressin; DDAVP) should also be established. Pre‐implantation genetic diagnosis as well as antenatal genetic diagnosis (via chorionic villus sampling) for haemophilia genotypes are available in Australia and can be considered in families where the genetic mutation responsible for the haemophilia has been identified.

Special considerations may need to be made for women with inherited bleeding disorders when undergoing invasive in vitro fertilisation procedures, such as oocyte retrieval, that carry a risk of bleeding. Management is multidisciplinary and includes the fertility specialist, haematologist and anaesthetist. Factor replacement and/or tranexamic acid around the time of procedure may be indicated. Consideration should be given to the most appropriate location in which such procedures should occur.^21^, ^22^

Women with rarer bleeding disorders, such as fibrinogen or factor XIII deficiency, may be at increased risk of recurrent fetal loss (Box 1). Such women may benefit from prophylactic therapy (Box 3).^23^

**Box 3:** Pre‐procedural administration of clotting factor products in women with factor levels below reference interval* 1, 2, 6, 13, 14, 16, 17, 24

Disorder	Product	Timing of treatment before procedure†	Duration of factor treatment after delivery	Further dose required for epidural/spinal catheter removal?
Haemophilia A	Recombinant factor VIII	0–2 hours	≥ 3 days or ≥ 5 days‡ (daily or twice daily)	Yes, if prior dose > 8 hours ago
Haemophilia B	Recombinant factor IX	0–2 hours	≥ 3 days or ≥ 5 days‡ (daily)	Yes, if prior dose > 8 hours ago
Factor XI deficiency	Factor XI concentrate (plasma derived); fresh frozen plasma in an emergency if factor XI concentrate unavailable	0–8 hours	1–3 days (every 1–2 days)	No, if prior dose < 36 hours ago
von Willebrand disease				
Type 1	VWF containing factor VIII concentrate	0–4 hours	1–3 days (daily)	Yes, if prior dose > 12 hours ago
Type 2§	VWF containing factor VIII concentrate	0–4 hours	1–3 days (daily)	Yes, if prior dose > 12 hours ago
Type 3	VWF containing factor VIII concentrate	0–2 hours	≥ 3 days or ≥ 5 days‡ (daily or twice daily)	Yes, if prior dose > 8 hours ago

VWF = von Willebrand factor. * Women with factor VIII or IX levels below 50 IU/dL or the lower limit of the reference interval (note that laboratory reference intervals vary based on methodology), or VWF levels < 30–50 IU/dL, at physician's discretion. ^†^ Including delivery, insertion of epidural or spinal catheter, chorionic villus sampling or amniocentesis procedures. ^‡^ Continue treatment for ≥ 3 days following vaginal delivery and ≥ 5 days following caesarean delivery, unless otherwise stated. ^§^ Thrombocytopenia associated with type 2B von Willebrand disease can worsen during pregnancy. ◆

### During pregnancy

While it is important to recognise that women with mild haemophilia and other inherited bleeding disorders may have an increased risk of bleeding depending on factor levels, they usually require no specific therapy or factor replacement antenatally.

Women with factor VIII or factor IX levels < 40 IU/dL, or type 2 or type 3 von Willebrand disease may be at risk of bleeding and may require factor replacement for procedures and delivery. The lower their factor level or von Willebrand factor activity, the more likely they will be to require replacement.^10^, ^25^

Bleeding in women with factor XI deficiency is highly variable, and provision of replacement therapy should be individualised; however, it may be required if factor XI levels are < 15 IU/dL.^6^, ^17^, ^18^ Treatment of women with rare bleeding disorders during pregnancy should be individualised and guided by a haemophilia clinician as early as possible (Box 1).

### Fetal sex determination

For women who carry the mutation for haemophilia and are pregnant with a male fetus, there is a 50% chance the fetus will be affected by haemophilia; for a female fetus there is a 50% chance she will inherit the mutation for haemophilia. While most female fetuses will have normal or only mildly reduced factor levels, very rarely a female fetus may have moderately or severely reduced factor levels. Fetal sex determination using ultrasound and free fetal DNA in maternal circulation aids labour and delivery planning, and may also preclude the need for more invasive prenatal diagnostic testing such as chorionic villus sampling and amniocentesis if the fetus is female. In the case of unknown fetal haemophilia status, it should be assumed that the fetus has haemophilia, and the pregnancy and delivery should be managed accordingly.

### Preparation for labour and delivery

Factor replacement should be organised before delivery if levels remain below normal (Box 2). Risk factors for postpartum haemorrhage should also be reviewed at this time and a clear intra‐ and postpartum multidisciplinary care plan for both mother and neonate should be documented.^19^

### Treatment options

In women with factor levels < 50 IU/dL, or if clinically indicated, tranexamic acid should be considered to cover surgical or invasive procedures.^6^ Following miscarriage, it should be continued until bleeding settles.^6^

DDAVP is a synthetic analogue of vasopressin that increases the plasma levels of von Willebrand factor and factor VIII by endothelial release. DDAVP can be used antenatally to raise factor VIII and von Willebrand factor plasma levels two‐ to sixfold during pregnancy in women with haemophilia A and type 1 von Willebrand disease;^6^, ^10^, ^19^, ^26^, ^27^, ^28^ it has a category B2 safety warning for use in pregnancy in Australia^29^ but does not cross the placenta at detectable levels.^28^, ^30^ with peak levels at 30–90 minutes after infusion, although there is considerable inter‐individual variation.^27^, ^28^, ^30^

Importantly, in most patients with type 1 von Willebrand disease who would respond to DDAVP, factor levels will have increased during pregnancy, but in patients with type 2 and type 3 von Willebrand disease, who have the greatest need for elevation of von Willebrand factor levels, the response to DDAVP is generally poor.^27^ As such, if treatment is needed, administration of von Willebrand factor‐containing concentrates is recommended in the antenatal treatment of von Willebrand disease, although DDAVP may be a suitable alternative in women who are carriers of haemophilia A.

It is important to note that DDAVP can stimulate uterine contraction and cause premature labour, as well as hyponatraemia.^28^, ^29^, ^30^ It has an antidiuretic effect, and fluids should be restricted to 1 L for 24 hours after use and electrolytes should be monitored.^6^

In women with Glanzmann thrombasthenia or Bernard–Soulier syndrome, blood and platelet transfusion should be avoided wherever possible because of the risk of sensitisation to antigens not present on their own platelets^6^, ^7^, ^8^ (Box 1). No treatment is required antenatally, but the presence of anti‐human leukocyte antigen and anti‐platelet antibodies should be assessed before delivery. If antibodies to fetal antigens are present, the delivery should be managed appropriately.

## Labour and delivery

Women with inherited bleeding disorders should be referred to a specialist haemophilia clinician, as individualised and specific treatment protocols must be followed. Ideally, women with severe bleeding disorders, or who are at risk of delivering a boy with haemophilia, should be managed jointly by an obstetric unit with facilities for caring for high risk infants and a haemophilia treatment centre.

The mode of delivery should be determined by obstetric indications. Spontaneous vaginal delivery, without instrumentation, is a suitable option for many women with inherited bleeding disorders, based on obstetric advice.^31^ It is important to note that an inherited bleeding disorder in the mother or fetus, by itself, is not an indication for delivery by lower segment caesarean section. Although lower segment caesarean section is an alternative option for delivery, it does not eliminate the risk of intracranial haemorrhage in the neonate, and elevates the risk of bleeding and factor replacement requirements of the mother.^6^, ^10^ Instrumental deliveries should be avoided because of the increased risk of intracranial haemorrhage. However, where an instrumental delivery is deemed unavoidable, a forceps delivery by an experienced accoucheur is preferred over vacuum extraction.^6^, ^10^, ^31^, ^32^, ^33^ Use of fetal scalp blood sampling, mid‐cavity manipulation, scalp electrodes and a prolonged labour should also be avoided if possible.^6^, ^10^, ^33^

Tranexamic acid 1000 mg, orally or intravenously, should be given to all women with an inherited bleeding disorder as close to the time of delivery as possible.^34^, ^35^ The dose should be repeated at 4 hours if there is postpartum haemorrhage.^34^, ^35^ If needed, factor replacement should be given to the mother as close to the time of delivery as possible, but it should be noted that this does not normalise the baby's factor levels. In haemophilia A and B and von Willebrand disease, factor replacement therapy, if required, should be administered so that levels are maintained above 50 IU/dL (ie, in the normal range) for labour and delivery. DDAVP has poor efficacy in type 2 and type 3 von Willebrand disease but may be used in carriers of haemophilia A.

In patients with severe platelet disorders, platelets (human leukocyte antigen matched if available) should be transfused at the time of delivery. Lower segment caesarean section should be offered to women with severe platelet function disorders.^6^, ^36^

## Obstetric anaesthesia

There are few studies reporting outcomes following the use of epidural or spinal anaesthesia in patients with inherited bleeding disorders and no guidelines that cover this topic comprehensively.

Patients with inherited bleeding disorders are at an increased risk of spinal haematoma.^6^, ^37^, ^38^ However, if coagulation factor levels are in the normal range, or supported and maintained in the normal range, then regional anaesthesia is not absolutely contraindicated.^1^, ^2^, ^6^, ^37^, ^38^, ^39^, ^40^, ^41^

It is recommended that factor VIII, factor IX and von Willebrand factor levels be maintained in the normal range (> 50 IU/dL) for epidural catheter insertion, the duration of catheter placement, catheter removal and for 12 hours (mild bleeding disorder) to 24 hours (moderate to severe bleeding disorder) after catheter removal (Box 3). If levels are < 50 IU/dL, epidural or spinal anaesthetic modalities should only be considered for use in close consultation with a senior anaesthetist and haematology team.

Alternative forms of analgesia and anaesthesia are available and may be more appropriate, depending on the context. In particular, patients with severe hereditary platelet functions, such as Glanzmann thrombasthenia and Bernard–Soulier syndrome, in general should not receive neuraxial anaesthesia.^6^, ^36^

## Postpartum care

To reduce the risk of postpartum haemorrhage and surgical bleeding, factor levels should be maintained in the normal range for ≥ 3 days after vaginal delivery and for ≥ 5 days after caesarean delivery.^1^, ^2^, ^6^, ^14^, ^17^ Factor levels should be monitored closely in the postpartum period, even in women whose levels normalised during pregnancy.^13^, ^16^, ^24^ Women who have low factor VIII, factor IX or von Willebrand factor levels after delivery are at continued risk of postpartum haemorrhage and should be advised to report symptoms. In the event of caesarean delivery, insertion of an intra‐abdominal drain in order to detect bleeding should be considered. Tranexamic acid is encouraged postpartum until lochia is minimal.

To minimise the risk of thrombosis, excessively high levels of factor VIII and IX should be avoided. Thromboprophylaxis should be considered in women with thrombotic risk factors whose factor levels have been normalised physiologically or therapeutically, especially in patients with von Willebrand disease.

## Postpartum haemorrhage

Postpartum haemorrhage is defined as blood loss > 500 mL; primary postpartum haemorrhage occurs within the first 24 hours and secondary postpartum haemorrhage occurs after 24 hours and before 6 weeks.

Obstetric causes are the most common reason for postpartum haemorrhage, and causes other than haemophilia should also be considered. Causes of primary postpartum haemorrhage include uterine atony, retained placental tissue,^42^ trauma to the female reproductive tract, and coagulopathy. Causes of secondary postpartum haemorrhage include retained placental tissue and infection. Women with low factor levels and von Willebrand disease have a significantly higher risk of both primary and secondary postpartum haemorrhage.^1^, ^2^, ^6^, ^17^, ^18^, ^19^, ^20^, ^24^, ^43^, ^44^

The risk of postpartum haemorrhage can be reduced by the active management of the third stage of labour.^45^ This includes administration of a prophylactic oxytocic agent within 2 minutes of the baby's birth to induce uterine contraction, immediate clamping and cutting of the cord to enhance placental separation, and placental delivery by controlled cord traction.^46^

Additionally, women with low factor levels should be identified as being at risk of postpartum haemorrhage and at least one large bore (16G) intravenous cannula should be inserted; up‐to‐date coagulation factor studies, full blood count and group and hold testing should be conducted.

Intrapartum management should be aimed at reducing the risk of postpartum haemorrhage (eg, labour should not be allowed to become prolonged). In addition to active management of third stage, there should be a low threshold for 40 IU oxytocin infusion (or use of other agents that facilitate uterine tone). Any trauma should also be promptly identified and repaired.

Management of postpartum haemorrhage should follow local guidelines;^47^ however, in women with early postpartum haemorrhage associated with low factor levels, factor replacement therapy or, in some instances, DDAVP (in carriers of haemophilia A or women with type 1 von Willebrand disease) may be required.^1^, ^2^, ^6^, ^19^, ^20^ DDAVP is not recommended for use in breast feeding mothers as it is transferred to breast milk (MIMS Online; http://www.mims.com.au).

Should late or secondary postpartum haemorrhage occur, first line management includes tranexamic acid,^6^, ^19^, ^35^ which is safe in breastfeeding mothers and is classed as category B1 for use in pregnancy in Australia.^29^ The oral contraceptive pill and, in the longer term, a levonorgestrel‐releasing intrauterine device are alternative therapies.^48^ Retained placental tissue and endometritis need to be excluded.

## Infants at risk of a severe bleeding disorder

Testing of cord blood for inherited bleeding disorders is recommended by AHCDO and is useful for excluding severe disease.^6^ However, its value in milder disease (particularly mild haemophilia B) is less certain, and results should be confirmed by peripheral blood testing. Factor IX is physiologically lower at birth and increases over the first 6–12 months of life; infants at risk of haemophilia B will therefore need retesting at a later stage to define baseline factor IX levels.^49^, ^50^ Other vitamin K‐dependent clotting factors and factor II, VII, IX, X and XI levels may be physiologically low in neonates.

All neonates at risk should be carefully observed for signs of intracranial haemorrhage, and transfontanelle ultrasonography should be considered soon after birth. Any neonate with bleeding should be managed in consultation with a haemophilia physician. Because intracranial haemorrhage may be delayed (median time after delivery is 4.5 days), mothers should be made aware of potential symptoms, such as vomiting, seizures and poor feeding, and be advised how to seek help if concerned.^51^

All neonates with an identified inherited bleeding disorder should be examined by a paediatrician and referred to the appropriate haemophilia treatment centre. It is important to note that even in neonates known to have a severe bleeding disorder, prophylactic factor replacement therapy should not be routinely given and may be associated with an increased risk of inhibitor development in children with haemophilia.

Inhibitor development is a serious complication for patients with haemophilia. It results from an immune‐mediated response that inhibits factor replacement, thus preventing control of a bleeding episode.^3^, ^6^, ^52^ Patients with inhibitors require the use of bypassing agents to achieve haemostasis.^53^ While inhibitors remit spontaneously in some patients, many require an intensive regimen of immune tolerance, which has an average success rate of 70%. Moreover, the use of prophylactic recombinant factor VIIa has not been shown to improve clinical outcomes.^3^

The risk of cranial haemorrhage is also increased in neonates with severe forms of von Willebrand disease^1^, ^2^ but is very rare in infants with factor XI deficiency.^18^ The risk of intracranial haemorrhage is about 2.5% in newborns with severe haemophilia, and the risk of extracranial haemorrhage is about 3.7%.^6^, ^10^, ^33^ In haemophilia, pre‐delivery ultrasound determination of fetal sex is useful, because female infants do not ordinarily have an elevated risk of cranial haemorrhage.

In general, intramuscular injections should be avoided until after the results of cord blood factor levels are available. Neonatal heel‐prick screening (Guthrie test) should still be carried out with firm pressure applied afterwards, to allow early identification of phenylketonuria, congenital hypothyroidism and cystic fibrosis. Vitamin K should be routinely administered, orally (or subcutaneously if required),^6^ and all three doses should be given, to avoid the risk of intramuscular haematoma. All infants, including those already known to have bleeding disorders, should be immunised for hepatitis B (administered subcutaneously or intradermally).^3^, ^6^

## Management of bleeding in neonates

Urgent liaison with a paediatric haemophilia treatment centre should be sought. Neonates known (or suspected) to have haemophilia A or B, and who have evidence of either intracranial bleeding or severe bleeding elsewhere, should receive immediate factor replacement with recombinant factor VIII or IX, respectively, to obtain plasma factor levels of 100 IU/dL in accordance with AHCDO guidelines and the product information.^54^

## Competing interests

No relevant disclosures.

## Provenance

Not commissioned; externally peer reviewed.
